# Supplementary material for: Non-Linear Response of Alpha and Beta Diversity of Taxonomic and Functional Groups of Phytoplankton to Environmental Factors in Subtropical Reservoirs
Source: Microorganisms. 2024 Jul 29;12(8):1547. doi: 10.3390/microorganisms12081547 (PMC11356485; doi:10.3390/microorganisms12081547)
Supplement: Supplementary file 1 [file microorganisms-12-01547-s001.zip › microorganisms-3044571-supplementary.pdf]

Supplementary material:

Table S1 Information of the 23 studied reservoirs in Han River Basin

Reservoirs	Longitude	Latitude	Depth (m)	Volume (×10^4 m^3)	Rain collecting area (km^2)	Maximum depth (m)	Water surface area (km^2)
MHT	116°31'	24°46'	75.2	20400	7907	105	64.0
YT	115°36'	23°57'	28.4	16500	251	35	8.34
HS	115°42'	24°16'	5.70	11612	577	15	0.87
CT	116°7'	24°43'	36.8	17200	1990	71	5.97
DS	116°29'	23°59'	10.0	7497	27503	10	0.96
HT	115°53'	24°43'	28.3	5230	140	46	2.60
MX	115°56'	24°23'	15.4	5100	350	24	5.00
FS	115°48'	24°33'	13.6	2388	53	41	1.59
DB	116°22'	24°52'	29.0	2213	68	45	2.02
HZP	116°14'	24°44'	24.5	1158	18	46	0.53
WG	115°43'	24°27'	16.6	2225.8	56	24	1.17
YQ	115°54'	23°54'	17.3	1860	37	33	0.68
GT	115°51'	23°49'	28.3	1328	41	56	0.35
HSY	115°47'	24°11'	12.2	1289	33	22	1.21
DFH	115°41'	23°54'	17.4	1408	29	28	10.0
SHB	116°36'	24°24'	10.2	2234	1603	12	0.40
SB	115°45'	24°15'	6.30	1006	102	/	0.68
QX	116°37'	24°35'	21.4	8470	9157	52	0.99
QLS	116°14'	24°12'	50.0	4864	94	66	1.08
FH	116°41'	23°57'	25.2	5990	164	29	0.42
GS	116°45'	23°40'	19.0	4611	88	25	2.67
FX	116°41'	23°57'	11.0	3270	46	60	1.23
PX	116°42'	23°57'	8.0	1136	12	50	0.13

Table S2 Environmental types indicated by main functional groups of phytoplankton in the 23 studied reservoirs

Functional group	Representative species	Environment	Reservoirs
**SN**	*Cylindrospermopsis rackiborskii*	Warm and well-mixed body of water	GS
**S1**	*Limnothrix redekei*	Cloudy and well-mixed body of water	HSY, HS, WG, MX
$\mathbf{L_O}$	*Merismopedia tenuissima*	Medium - large reservoirs, nutrition stratification	YT
**B**	*Cyclotella meneghiniana*	Medium eutrophic water body	PX, DS
**Y**	*Cryptomonas ovata*	Still water	DB
**P**	*Melosira granulata*	Continuously mixed water	MHT
**MP**	*Pseudoanabaena limnetica*	Turbid and shallow water	SB
**M**	*Microcystis wesenbergii*	Stable eutrophic water	SB
**N**	*Staurastrum gracile*	Mesotrophic mixed water	FS
**J**	*Scenedesmus quadricanda*	Nutrient-rich shallow water body	DFH, DB
**F**	*Nephrocytlum agardhianum*	Mesotrophic reservoir	FH
**X1**	*Monoraphidium komarkovae*	Highly mixed water	YT
**E**	*Dinobryon divergens*	Oligotrophic body of water	GS
**D**	*Synedra ulna*	Turbid	/

Table S3 Mantel test between environmental distances and beta diversity using the analysis of Bray-Curtis dissimilarity matrices

	Wet season		Dry season	
	**R**	***p***	**R**	***p***
Functional group & Environmental variables	0.214	0.007	0.057	0.215
Species & Environmental variables	0.133	0.049	0.013	0.408

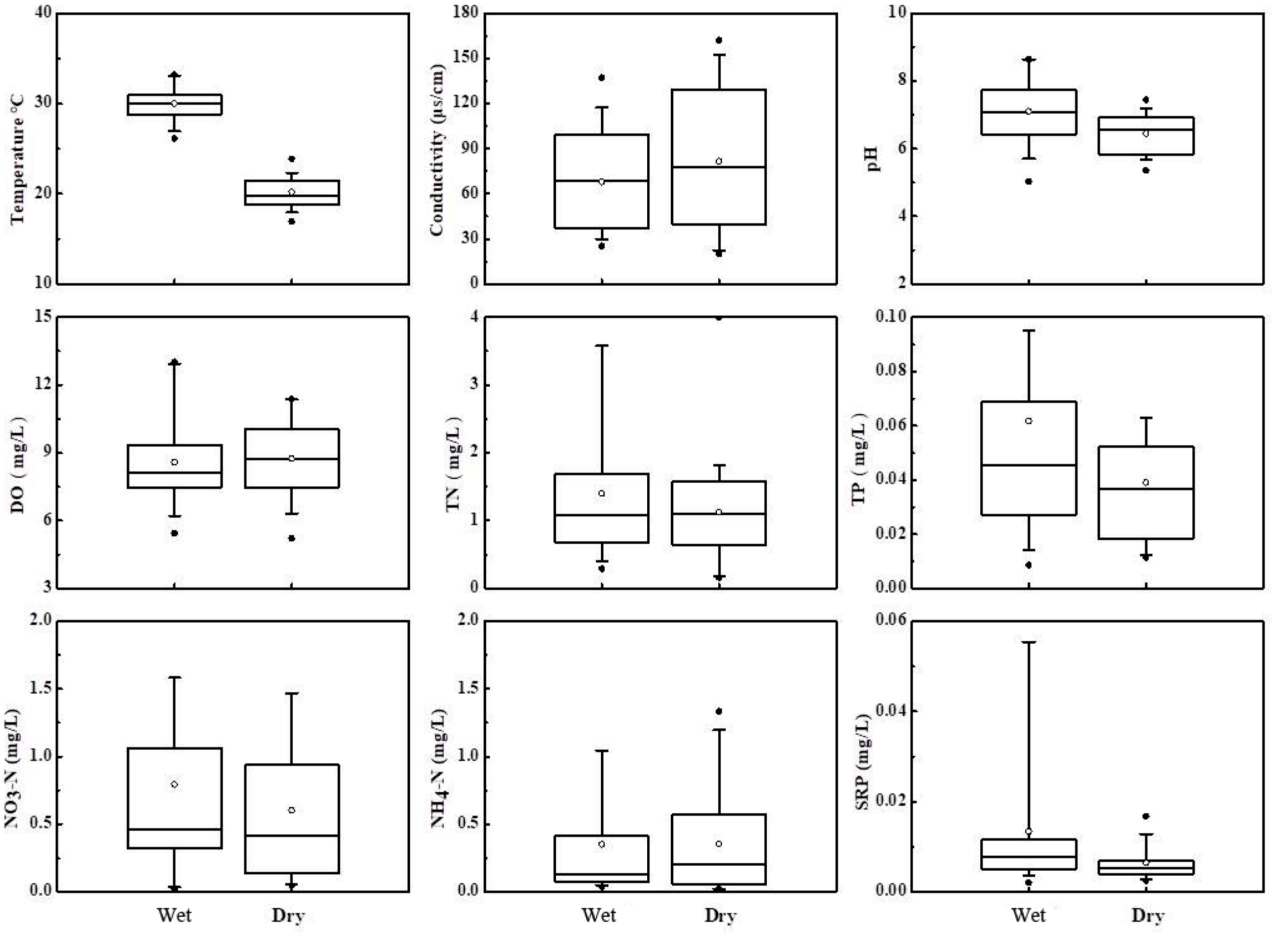

Figure S1 Physicochemical variables in the studied reservoirs in wet and dry seasons

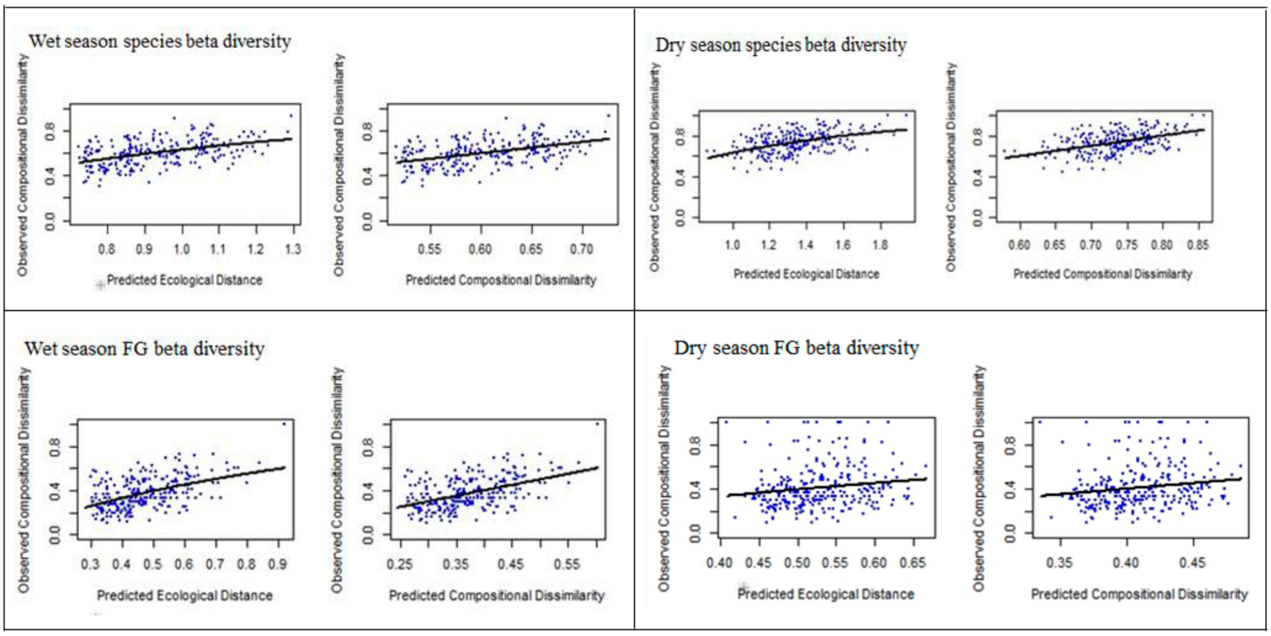

Figure S2 Comparative response of species and functional groups to ecological distances across sampling points: GDM-predicted versus observed in wet and dry seasons
